# Cellular signaling pathways modulated by influenza infection in HEK293 cells

**DOI:** 10.1186/1753-6561-9-S9-P9

**Published:** 2015-12-14

**Authors:** Aziza P Manceur, Amine A Kamen, Emma Petiot, Chun F Shen, Sven Ansorge

**Affiliations:** 1 Vaccine Program, Human Health Therapeutics, National Research Council, Montréal, Québec, H4P 2R2, Canada; 2 Department of Bioengineering, Macdonald Engineering Building, McGill University, Montréal, Québec, H3A 0G4, Canada; 3 Laboratoire de Virologie & Pathologie Humaine (VirPath), Université Claude Bernard Lyon 1, 69372 Lyon Cedex 08, France

## Background

Cell-culture based vaccines are a valuable alternative to egg-produced vaccines. The equivalent of 1500 influenza vaccine doses can be produced in a 1L bioreactor within 48 hours with HEK293 cells. The kinetics of production of H1N1 A/Puerto Rico/8/68 in HEK293 cells has been previously characterized by our group[1]; higher viral production is achieved when cells are infected at a multiplicity of infection (MOI) of 0.01 compared to an MOI of 1. Also, two cycles of infection take place: a first round of virus is produced at 8 hours post-infection (hpi) which then infect cells again and lead to a second viral exit at 16 hpi.

Infection triggers a cascade of intracellular responses which contributes to viral replication, but eventually leads to cell death. Most studies focus on one pathway at a time, and aim at limiting the extent of the infection through the use of inhibitors. Our goal however is to increase viral yield and quality. The main objective is therefore to understand and manipulate the molecular events taking place at the cellular level upon influenza infection and replication, with particular attention to key time points corresponding to viral production and exit. Signaling pathways examined include the Akt, mTOR and ERK pathway, and their activation levels were determined by measuring their phosphorylation states.

## Materials and methods

HEK293 cells were grown in suspension in serum free media (SFM4Transfx-293, HyClone). Cells at a density of 2E06 cells/ml were infected at an MOI of 0.01, in the presence of 1 μg/ml trypsin-TPCK, with H1N1 A/Puerto Rico/8 (H1N1 PR/8) or H3N2 A/Aichi/8/68 (H3N2 Aichi). Non-infected cells, but treated with trypsin-TPCK were used as a negative control. Samples were collected at 0.5 to 42 hpi, fixed with 2% paraformaldehyde, permeabilized and stored in methanol at -80oC until analysis. Phospho-specific antibodies (Cell Signaling) were used to measure phospho-Akt (S473), phospho-mTOR (S2448) and phospho-Erk (Thr202-Tyr204) using flow cytometry. The presence of influenza virus was simultaneously assessed by staining hemagglutinin (HA), the main surface protein of influenza. Cell viability was monitored throughout the infection period with an automated cell counter (Cedex HiRes, Roche). Viral production was quantified by measuring HA concentration using the dot-blot technique and an anti-HA antibody developed in house. The infectious titer was determined by TCID50 in MDCK cells.

## Results

**Figure 1:**
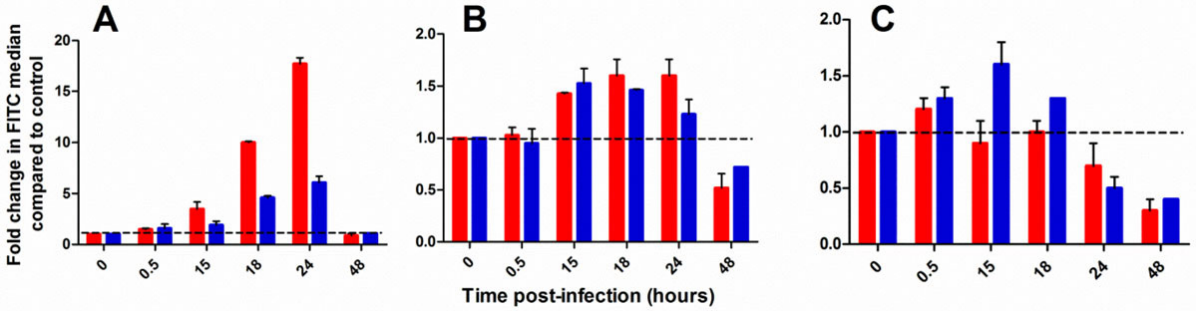
**Profiles of activation of phospho-Akt (A), phospho-mTOR (B) and phospho-ERK (C)**. The phosphorylation state of each kinase was detected with a phospho-specific FITC-labelled antibody. Results are presented as fold difference compared to the non-infected control. Average ± SD (n = 2). The dashed line represents no change in the infected samples compared to control (a fold change of 1). Legend: H1N1 PR8 is shown in red, and H3N2 Aichi is shown in blue.

Cell viability remained above 97% during the first 24 hpi, and declined to less than 50% by 42hpi. Influenza infection with H1N1 PR/8 highly activated Akt with an 18-fold increase in phosphorylation at 24hpi. H3N2 Aichi also activated Akt but to a lesser extent with a 6-fold increase in phosphorylation at 24hpi (Figure 1A). These results suggest that signaling pathways are differentially modulated depending on the strain of influenza virus. Akt was reported to be activated during viral replication in order to prevent premature apoptosis [2-6]; the viral Non-Structural Protein 1 (NS1) activates the Akt pathway in the late stages of infection which inhibits Caspase-9 and results in anti-apoptotic signaling. NS-1 is strain specific and could explain why Akt is more highly activated by H1N1 PR/8 than by H3N2 Aichi.

In parallel, the phosphorylation of mTOR was increased during the first 24hpi but remained stable, and was similar for the two strains of influenza examined (Figure 1B). Finally, ERK was initially activated by infection with H3N2 Aichi but not with H1N1 PR/8, and decreased at 24hpi with both strains (Figure 1C).The profile of activation of each kinase reflects different cellular events caused by viral infection: mTOR is solicited during protein synthesis while Erk is involved in export of viral ribonucleoproteins from the nucleus to the cytoplasm [7-9].

Next, in order to increase viral yield, we first focused on modulating Akt in cells infected with H1N1 PR/8.Based on the profile of activation of Akt, small molecules were added to either inhibit or activate Akt at time of infection (T.O.I) which corresponds to viral entry, or 15 to 17hpi which coincides with viral exit. Viral yield was measured by quantifying the total amount of HA, the main surface protein produced by influenza viruses, and by evaluating the infectious titer in a TCID50 assay. Conditions in which Akt is inhibited at T.O.I. or activated at 17hpi, resulted in a significant increase in HA concentrations by 2 fold and in infectious viral titer by 1 log (Table 1). In contrast, inhibiting Akt at a later time in the infection process, or activating Akt at T.O.I. did not improve viral yield (Table 1). This finding highlights the importance of considering the kinetics of activation of molecular switches when designing feeding strategies.

**Table 1:** Effect of small molecules used to modulate Akt activity at different time points after infection with H1N1 PR/8

	Control	Akt activator (T.O.I)	Akt activator (17hpi)	Akt inhibitor (T.O.I.)	Akt inhibitor (17hpi)
**HA concentration (μg/ml)**	18.4 ± 0.8	2.9 ± 0.6	35.1 ± 5.2	31.2 ± 3.9	21.0 ± 4.4
**TCID50 (IVP/ml)**	1.1E10	8.6E09	3.6E11	5.4E11	9.8E09

## Conclusions

We have systematically investigated multiple signaling pathways using flow cytometry, with particular attention to time points corresponding to viral entry and exit. An understanding of the timing of these events will facilitate the selection of small molecules to control signaling pathways and we have first evidence that this will lead to an improved feeding strategy. As a proof of principle, we show that modulation at Akt at strategic time points significantly increases viral yield. Other important signaling molecules that will be examined include PKC, NFkB and P53. Based on the data thus obtained, a cocktail of small molecules can be prepared and added to the culture at the most appropriate time in order to improve the yield and the quality of the virus generated for vaccine production.
